# Zinc-modified phosphate-based glass micro-filler improves *Candida albicans* resistance of auto-polymerized acrylic resin without altering mechanical performance

**DOI:** 10.1038/s41598-022-24172-y

**Published:** 2022-11-14

**Authors:** Myung-Jin Lee, Min-Ji Kim, Utkarsh Mangal, Ji-Young Seo, Jae-Sung Kwon, Sung-Hwan Choi

**Affiliations:** 1 Department of Dental Hygiene, Division of Health Science, Baekseok University, Cheonan, Republic of Korea; 2 Department of Orthodontics, Institute of Craniofacial Deformity, Yonsei University College of Dentistry, 50-1 Yonsei-Ro, Seodaemun-Gu, Seoul, 03722 Republic of Korea; 3 BK21 FOUR Project, Yonsei University College of Dentistry, Seoul, Republic of Korea; 4 Department and Research Institute of Dental Biomaterials and Bioengineering, Yonsei University College of Dentistry, 50-1 Yonsei-Ro, Seodaemun-Gu, Seoul, 03722 Republic of Korea

**Keywords:** Dental biomaterials, Removable prosthodontics

## Abstract

Colonization of auto-polymerized acrylic resin by pathogenic *Candida albicans* is a common problem for denture users. In this study, zinc-modified phosphate-based glass was introduced into an auto-polymerized acrylic resin at concentrations of 3, 5, and 7 wt.%. The mechanical or physical properties (flexural strength, elastic modulus, microhardness, and contact angle), surface morphology of the resultant materials, and the antimicrobial effect on *C. albicans* were investigated. There were no statistical differences in the mechanical properties between the control and the zinc-modified phosphate-based glass samples (*p* > 0.05); however, the number of *C. albicans* colony-forming units was significantly lower in the control group (*p* < 0.05). Scanning electron microscopy revealed that *C. albicans* tended not to adhere to the zinc-modified-phosphate-based glass samples. Thus, the zinc-modified materials retained the advantageous mechanical properties of unaltered acrylic resins, while simultaneously exhibiting a strong antimicrobial effect in vitro.

## Introduction

The rapid growth of the elderly population has resulted in a corresponding increase in the number of denture users^1^. The base of dentures is often made of acrylic resin, especially poly(methyl methacrylate) acrylic (PMMA)^2^, an easy-to-use, moldable, and affordable material that offers satisfactory aesthetics and excellent biocompatibility. However, PMMA and other denture base resins are susceptible to microbial colonization in the oral environment^3^.

The absence of ionic charges in the auto-polymerized acrylic resin prevents protective saliva molecules from adhering to the surface of the denture and thus promotes the formation of biological membranes^4^. Hydrophobic interactions and mechanical attachment (in conjunction with local roughness, surface porosity, and poor hygiene) may also induce bacterial attachment. The denture base functions as a substrate for microbial adhesion and biofilm formation, thereby resulting in denture stomatitis. This may induce additional complications such as fungal infections, which are highly significant for elderly and immunosuppressed patients^5,6^.

*Candida albicans* is the primary pathogen in denture stomatitis, a widely recurring disease that affects approximately 11–67% of denture users^7,8^. *C. albicans* on the surface of acrylic resin used as a denture base material or oral epithelium is usually in a non-pathogenic state; however, it induces diseases in patients with weakened immunity as well as opportunistic infections^9^. The prevention or suppression of denture stomatitis is essential because the formation of *C. albicans* biofilms is associated with severe local and systemic infections in denture users^10^. *C. albicans* is released into saliva and may be subsequently aspirated into the lower respiratory tract, inducing pneumonia in the elderly^11^. Therefore, a method to optimize the antimicrobial properties of auto-polymerized acrylic resin is needed.

A 1–2% solution of chlorhexidine gluconate is used for the treatment of *C. albicans*-induced denture stomatitis; however, this treatment induces discoloration^12^. Conventional methods of treating oral candidiasis involving the use of dentures are generally effective for the removal of accumulated plaque but pose challenges for the elderly, especially those with disabilities or who require nursing care^13^. Antimicrobial agents are also effective in the control of dentures but induce toxic side effects and the development of resistant strains. This results in deterioration of the physical and mechanical properties of the dentures^3,14^. Therefore, research on inorganic metals that are effective in preventing fungal infections is being actively conducted^15,16^.

Recently, certain inorganic materials have been shown to exhibit broad-spectrum biocidal effects^17,18^. Various inorganic fillers have therefore been proposed for modifying the properties of acrylic-based resin^17,19^. Most fillers are intended to augment microbial resistance^20^. To eliminate *C. albicans*, both micro-fillers and nano-fillers have been proposed^21–23^. Among these inorganic materials, zinc ions in particular have been explored as an alternative to conventional antimicrobials in dental materials^24,25^. The aforementioned additives have shown variable microbial resistance, often observed to increase with increasing concentration of additives^21–23,26,27^. However, the antimicrobial effects of the additives are mostly associated with compromised mechanical properties^23^. Compared to Zn-dopped particles or zinc oxides, PBG glass releases ions slowly for a long period of time; therefore, its dissolution rate can be controlled. PBG glasses also exhibit a bioactivity in the glass composition range^28^.

Most microparticles change the mechanical properties of the acrylic resin to which they are added because they are incompatible with its matrix structure^6^. Similar undesired changes in mechanical properties have also been observed with the use of nanoparticles, mainly due to particle agglomeration^29^. Although silanization is believed to improve homogeneity, a matrix-compatible micro-filler to improve the properties of acrylic-based denture resins has not been found^26,30^.

Amongst the different biocompatibile fillers, glass based fillers are preferred additives in the resin matrix. By varying the proportion and type of the constituents, the stability, durability, and biological activity of the glass can be optimized. Phosphate-based glasses (PBGs) with properties such as high thermal expansion, optimal UV transparency, and favourable glass transition temperature have been incorporated with PMMA type resin matrices^31^. Although the results have shown variable success, the underlying nature of the PBG having a composition-dependent chemical durability limits its application^32–35^. To this end, different dopants have been used to adjust the composition of the PBG, affecting its durability^31^. Herein, we propose composition modification with zinc. By integrating Zn into the PBG structure, the density of the formed powdered glass is increased. High density filler can be incorporated at a low weight percentage in the resin matrix while maintaining the desired effect. Therefore, the chemical durability of zinc-modified PBG (Zn-PBG) micro-filler can be beneficial for PMMA.

From these considerations, we propose the use of Zn-PBG microparticles synthesized by zinc modification of the phosphate-dominant bioglass. We examine the key mechanical properties of the acrylic resin, evaluate the ionic elution, and comprehensively evaluate the material’s resistance to *C. Albicans.*

## Materials and methods

### Glass preparation

To obtain glass powder, P_2_O_5_ (42 mol%; 99%, Sigma-Aldrich), CaO (25.2 mol%; 99.9%, Sigma-Aldrich), Na_2_O (16.8 mol%; 97%, Sigma-Aldrich), and ZnO (16 mol%; 99.9%, Sigma-Aldrich) powders were mixed in a tubular shaker–mixer (Model T2F, Glen Mills Inc., USA) for 60 min at 100 rpm. Each batch of powder was melted in an alumina crucible using an electric furnace (Lindberg, Asheville, NC) with a heating rate of 10 °C/min until 1100 °C. Subsequently, the melted glass was quenched at room temperature to obtain a glass cullet. This was followed by grinding in an alumina mortar and subsequent pulverization under dry conditions using a planetary mono-mill (Pulverisette-7; Fritsch, Idar-Oberstein, Germany) for 10 min at 350 rpm.

### Incorporation of Zn-PBG into auto-polymerized acrylic resin

A commercially available orthodontic acrylic resin (Ortho-Jet, Lang Dental Manufacturing Co. Inc.) was used according to the manufacturer’s instructions. The material was an auto-polymerized resin system. The Zn-PBG powder was homogeneously mixed with the acrylic-resin powder using a high-speed mixer (Speed Mixer, Hauschild, Hamm, Germany) at 3500 rpm for 2 min. The wt.% of the Zn-PBG was calculated to match the final concentration in the resin at various weight concentrations (3, 5, and 7 wt.%). The unmodified resin was used to fabricate control group specimens. The compositions are listed in Table 1. For all the specimens, the powder and liquid were mixed in a mass ratio of 3:2. The polymerization was performed at low-temperature (60 °C; this is comparable to the temperature of heat-activated resin) using an air press unit (4.0 bar, 15 min, Air Press Unit, Sejong Dental). Thereafter, all the specimens were polished with progressively increasing fineness from 800 to 2000 grit papers.

**Table 1:** Compositions of the control and experimental groups in this study.

Group	Auto-polymerized acrylic resin (wt.%)	Zn-PBG (wt.%)
Control	100	0
3 wt.% Zn-PBG	97	3
5 wt.% Zn-PBG	95	5
7 wt.% Zn-PBG	93	7

### Density, molar volume, and elemental composition of Zn-PBG powder

The density of the prepared Zn-PBG was evaluated using a pycnometer (AccuPyc II 1340; Micromeritics Instrument Co., Norcross, GA) and the molar volume was calculated using the measured density and molecular weight. Field-emission scanning electron microscopy (FE-SEM; Merin, Carl Zeiss, Oberkochen, Germany) in conjunction with energy-dispersive X-ray spectroscopy (EDS) was performed to determine the elemental composition.

### Flexural strength and elastic modulus

The flexural strength and elastic modulus were tested according to the International Standard ISO 20,795–1. Seven specimens were manufactured for each PMMA group with dimensions of 64 × 10 × 3.3 mm^3^. The prepared specimens were immersed in 10 mL of distilled water and stored at 37 °C for 24 h. Thereafter, all the specimens were loaded to fracture using a universal testing machine (Model 5942, Instron, Norwood, MA, USA) with a span length of 50 mm and a crosshead speed of 5 mm/min. The flexural strength σ and elastic modulus E were calculated from as follows:

$${\upsigma } = \frac{3Fl}{{2bh^{2} }} ,$$

$${\text{E}} = \frac{{Pl^{3} }}{{4bh^{3} d}} ,$$

where *F* is the maximum load, *l* is the distance between the supports (mm), *b* and *h* are the width and height of the specimens (mm) prior to water storage, *P* is the load at a point in the straight-line portion of the load/displacement curve, and *d* is the deflection at load *P* (mm).

### Microhardness

Three specimens (diameter, 10 mm; thickness, 2 mm) were prepared for each group. A total of 12 specimens were assessed via Vickers hardness testing (MMT-X, Matsuzawa Seiki Co., Tokyo, Japan), using a Vickers diamond indenter with load and dwell times of 50 g and 10 s, respectively. Three points were measured randomly for each specimen, and the mean value and standard deviation were calculated.

### Contact angle

The wettability of each specimen was determined via contact-angle analysis (SmartDrop, Femtobiomed Inc., Gyeonggi-do, Korea). Three samples from each group were considered (diameter, 10 mm; thickness, 2 mm). Distilled water (5 µL) was randomly dropped on the surface, and the contact angle was measured after 10 s of contact. This process was repeated three times.

### Scanning electron micrographs of the sample surface

Prior to measurement, all the manufactured specimens were sputter-coated using platinum to facilitate observation of the material surface. The SEM (Merin, Carl Zeiss, Oberkochen, Germany) images were obtained under an accelerating voltage of 15 kV and magnification 500 × .

### Standard preparation of fungal specimens

*C. Albicans* ATCC 10,231 was cultured at 37 °C in a yeast media (YM, Becton Dickinson and Co., Franklin Lakes, NJ, USA) for 24 h. A discoid specimen was prepared; thereafter, 1 mL of fungal suspension (1 × 10^8^ cells/mL) was placed on each disk in a 24-well plate and incubated at 37 °C for 24 h under a ≥ 95% relative humidity atmosphere in a temperature and humidity controlled chamber. The specimens were gently washed twice using phosphate buffered saline (PBS) to remove any mold after incubation.

### Morphology

*C. Albicans* specimens prepared in the standard way were placed in 2% paraformaldehyde–glutaraldehyde in 0.1 M PBS buffer (pH 7.4) for at least 30 min at room temperature. The specimens were post-fixed with 1% OsO_4_, which was dissolved in 0.1 M PBS for 2 h. Subsequently, they were dehydrated in ethanol, treated with isoamyl acetate, and subjected to critical-point drying (LEICA EM CPD300; Leuca, Wien, Austria). Thereafter, the specimens were subjected to Pt-ion coating (5 nm; ACE600; Leica). This was followed by examination and imaging via SEM (Merin, Carl Zeiss, Oberkoche, Germany) at 2 kV.

### Colony-forming units (CFUs)

*C. Albicans* specimens were prepared in the standard way. The attached fungi were harvested for 5 min in 1 mL of YM via sonication (SH-2100; Saehan Ultrasonic, Seoul, Korea). The procedure was adapted from a previous study^24,36^. The fungal cultures were diluted to obtain an optical density (OD_600_) of 0.4–0.6 using an optical density reader (Epoch, BioTek, Winooski, VT, USA). Subsequently, fungal solution of 1 × 10^8^ cells/mL of *C. Albicans* was added to the specimen. After 24 h, 100 µL of this fungal suspension was spread on YM agar plates and incubated at 37 °C for 24 h. Thereafter, the total number of colonies was calculated. Five specimens were made for each group, and the mean and standard deviation were calculated accordingly.

### Ion release

Disk-shaped samples were formed using a Teflon mold with a diameter and thickness of 10 and 2 mm, respectively. Five disks were fabricated from each group, and all the samples were stored in 5 mL of distilled water containing 15 mL conical tubes. The specimens were then stored at 37 °C for 24 h. The Ca, P, and Zn ions released from the specimens were detected using inductively coupled plasma-optical emission spectrometry (ICP-OES, Optima 8300, PerkinElmer, Waltham, MA, USA). The operating conditions were a radio frequency (RF) power of 1500 W, a plasma gas flowrate of 18 L/min, an auxiliary gas flow of 1.0 L/min, a plasma gas flow rate of 15 L/min, and a sample pumping rate of 1.2–1.8 mL/min.

### Statistical analysis

All statistical analyses were performed using IBM SPSS Statistics for Windows, Version 25.0 software (IBM Corp. Released 2017. IBM SPSS Statistics for Windows, Version 25.0. Armonk, NY: IBM Corp.). The level of significance was assessed at *p* < 0.05. The results of all groups were analyzed using a one-way analysis of variance followed by Tukey’s post hoc test.

## Results

### Analysis of the elemental composition of Zn-PBG powder

**Figure 1:**
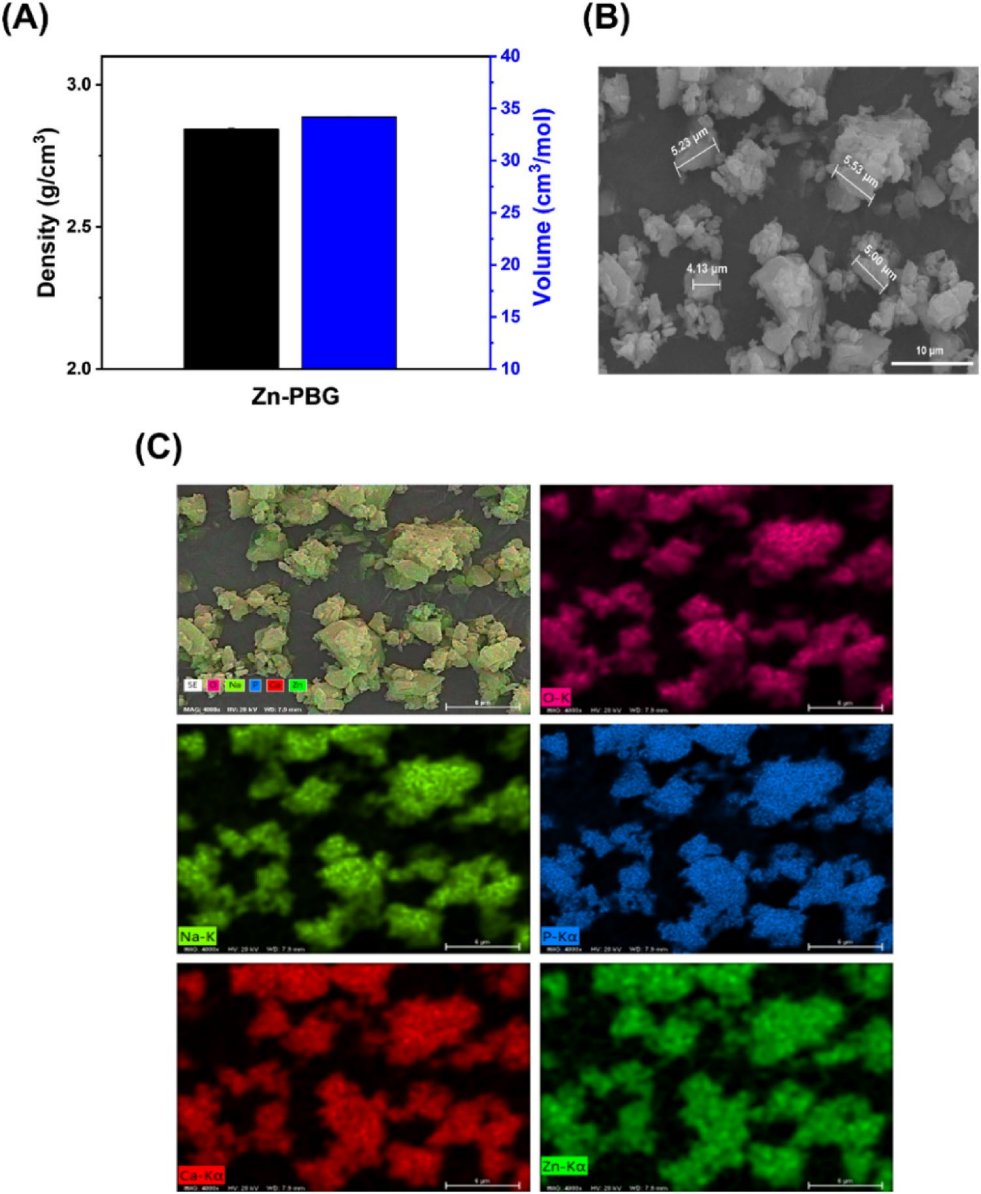
(**A**) Density and volume of Zn-PBG powder, (**B**) Energy-dispersive X-ray microscopy (EDS) images of Zn-PBG powder, and (**C**), EDS mapping images of various elements.

The density and molar volume of Zn-PBG were 2.84 g/cm^3^ and 34.18 cm^3^/mol, respectively, as shown in Fig. 1A. The morphology of the crushed Zn-PBG powder is shown in Fig. 1B. There are a range of sizes and aspect ratios, and the powders are irregular and somewhat aggregated. The granules of the powder size appear to be approximately 5 μm. In addition, the chemical distribution of phosphorus (P), calcium (Ca) sodium (Na), oxygen (O), and zinc (Zn) was fairly homogeneous (Fig. 1C).

### Flexural strength and elastic modulus

**Figure 2:**
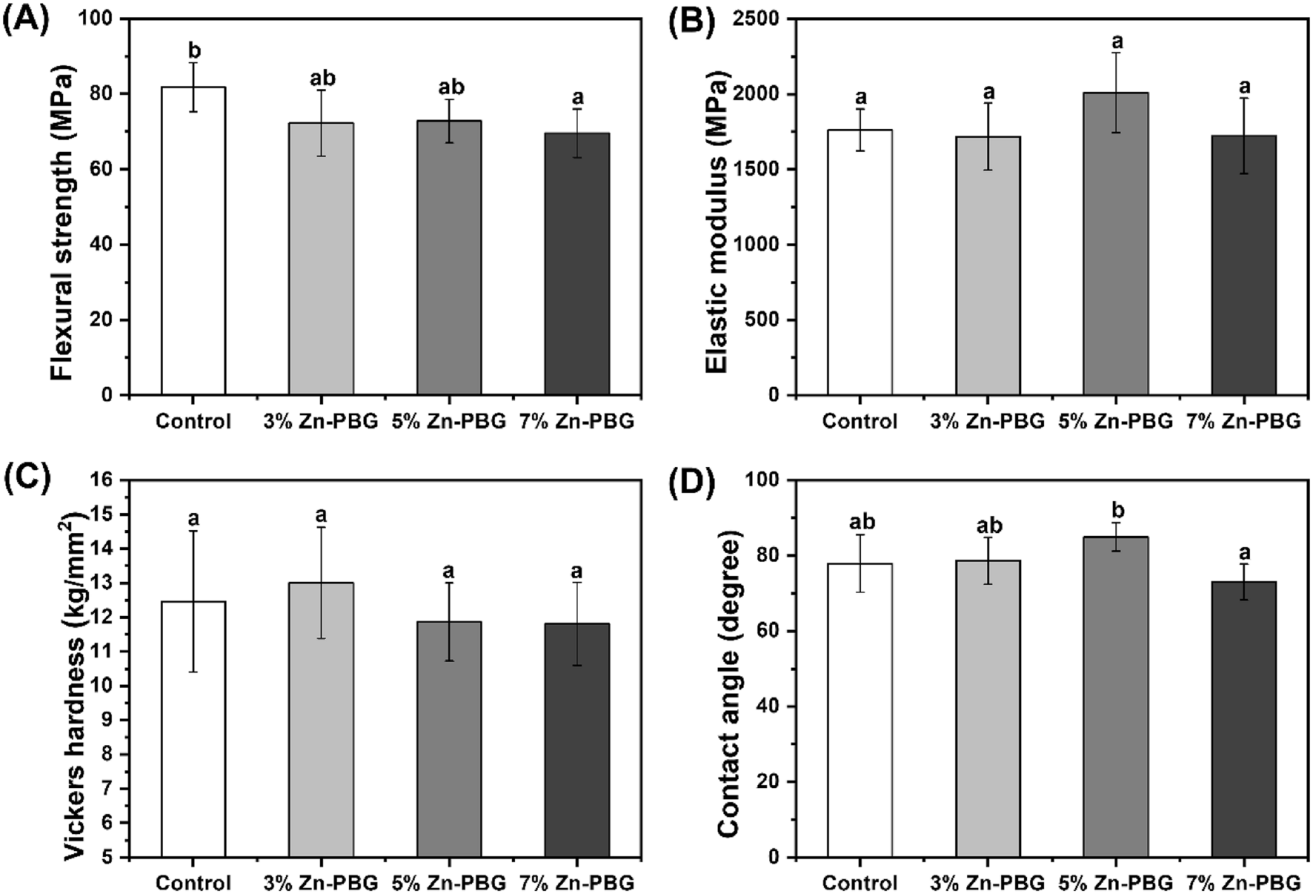
Physical and mechanical properties of different auto-polymerized acrylic resin specimens; each value represents the mean of seven measurements and the error bars show the standard deviation of the mean (mean ± standard deviation; *n* = 7). Different lowercase letters above a bar indicate a significant difference at *p* < 0.05. (**A**) Flexural strength. (**B**) Elastic modulus. (**C**) Microhardness. (**D**) Contact angle.

The measured flexural strength and elastic modulus are presented in Figs. 2A and B, respectively. The flexural strength increased with the amount of Zn-PBG, and all the groups met the 60 Mpa requirements of the ISO 20,795–1 standard. Although the flexural strengths of the control group (81.77 ± 6.55 Mpa), 3% Zn-PBG (72.27 ± 8.76 MPa), and 5% Zn-PBG (72.77 ± 5.79 MPa) did not show significant differences (*p* > 0.05), the flexural strength of the 7% Zn-PBG group (69.57 ± 6.40 Mpa) differed significantly from that of the control (*p* < 0.05). The elastic modulus of all the groups showed no significant difference with increasing Zn-PBG (*p* > 0.05).

### Microhardness

The microhardness results of the control (12.46 ± 2.06) and experimental groups (3% Zn-PBG: 13.00 ± 1.61, 5% Zn-PBG: 11.86 ± 1.13, and 7% Zn-PBG: 11.80 ± 1.21) are shown in Fig. 2C. There were no significant differences in the results across all the groups (*p* > 0.05). This indicated that the addition of Zn-PBG to PMMA did not have any effect on the surface microhardness.

### Contact angle

The measured contact angles for all the groups are shown in Fig. 2D. The 5% Zn-PBG group exhibited the highest contact angle (84.94 ± 3.73°). However, there were no significant (*p* > 0.05) differences in the results for the control (77.87 ± 7.62°) and 3% Zn-PBG (78.63 ± 6.20°) groups. The 7% Zn-PBG group exhibited the lowest contact angle (73 ± 4.73°); this was significantly different from the contact angle of the 5% Zn-PBG group (*p* < 0.05).

### SEM images of the sample surface

**Figure 3:**
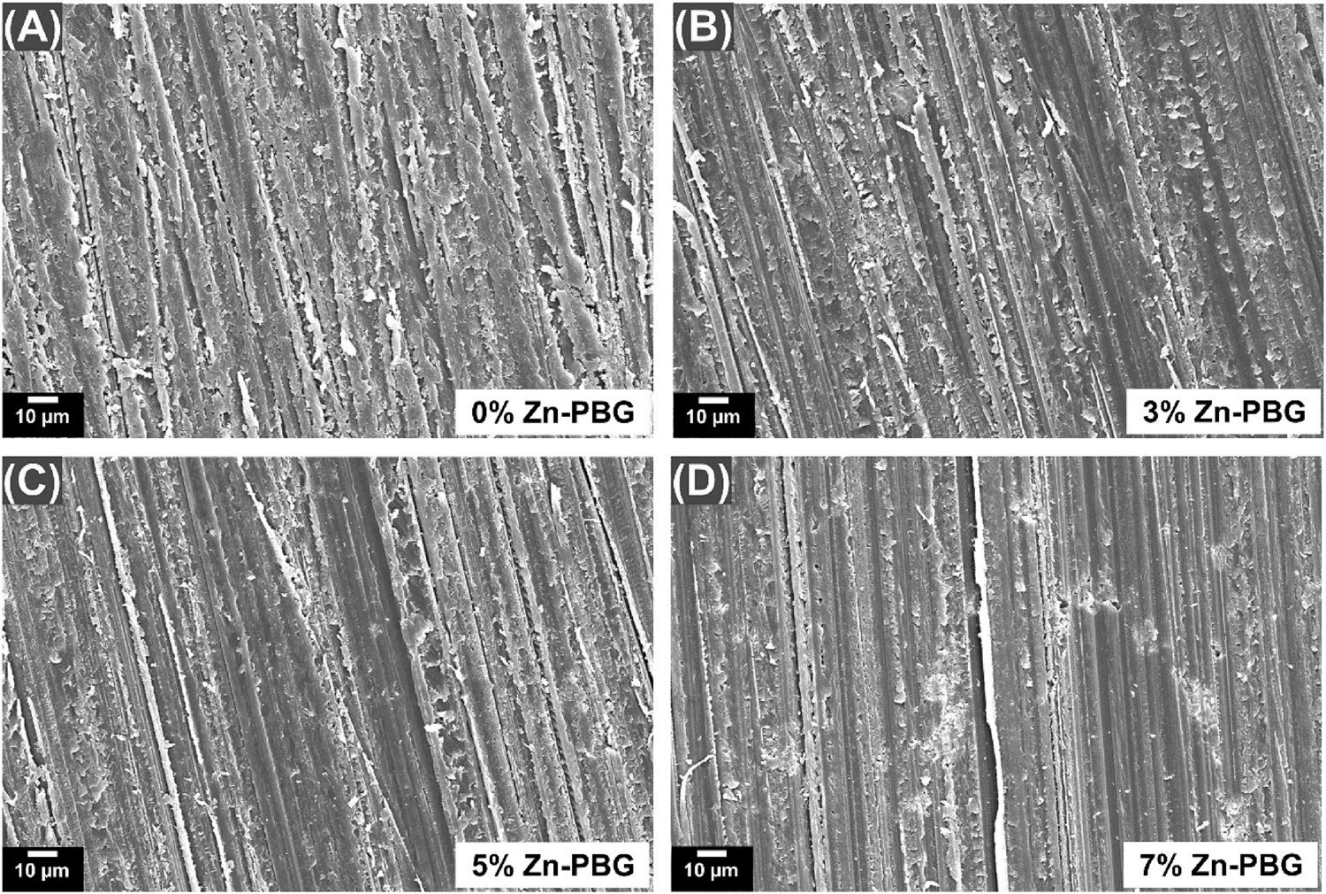
Scanning electron micrographs of the surfaces of various auto-polymerized acrylic resin samples used in this study at 500 × magnification: (**A**) control; (**B**) 3% Zn-PBG; (**C**) 5% Zn-PBG; (**D**) 7% Zn-PBG.

The SEM images of the auto-polymerized acrylic resin specimen were recorded for surface analysis (Fig. 3). There were no significant differences in the surface morphologies of each group. These results indicate that Zn-PBG did not affect the PMMA surface.

### Antifungal properties

**Figure 4:**
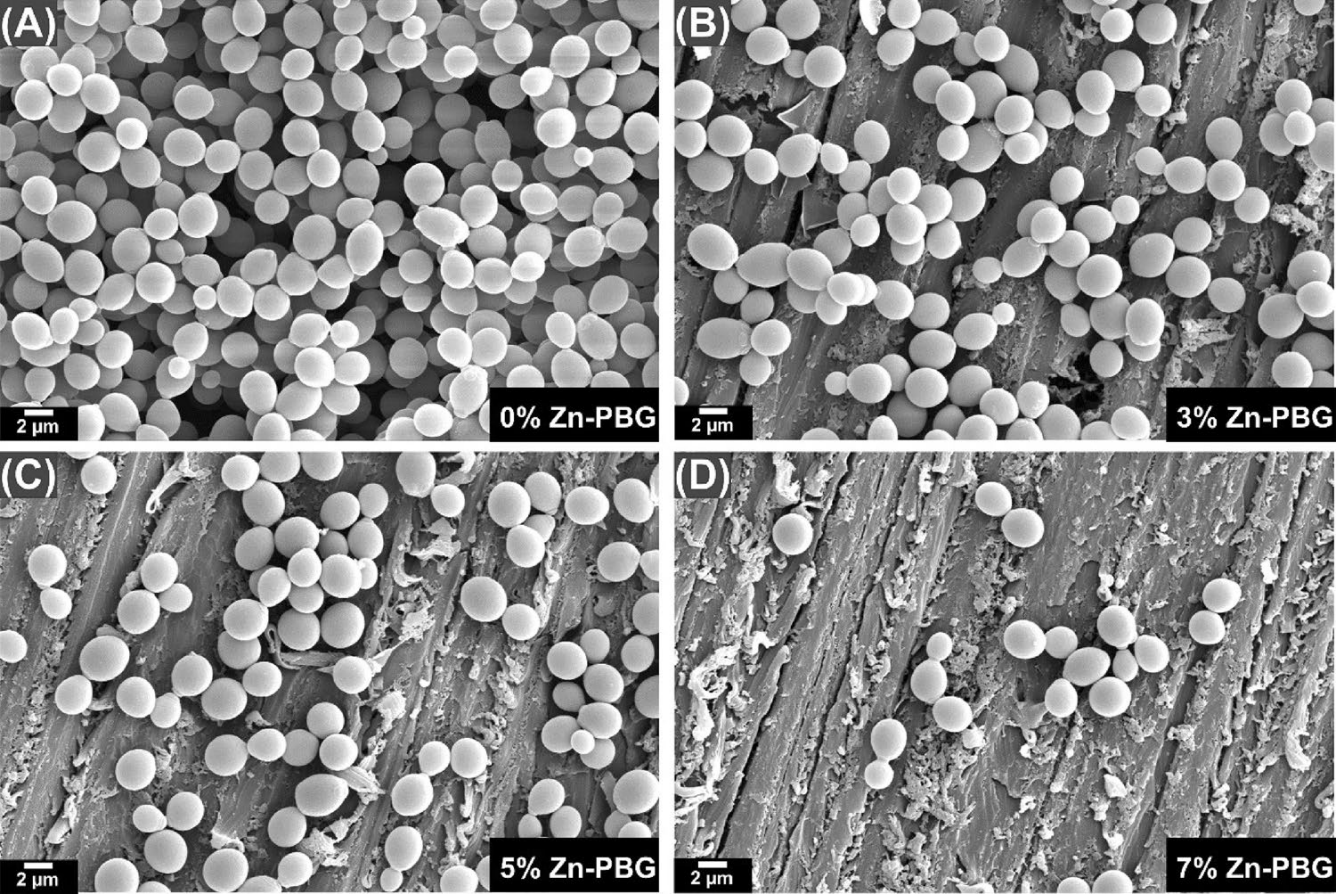
Representative scanning electron micrograph**s** of *Candida albicans* on the surfaces of various auto-polymerized acrylic resin samples: (**A**) control; (**B**) 3% Zn-PBG; (**C**) 5% Zn-PBG; (**D**) 7% Zn-PBG.

**Figure 5:**
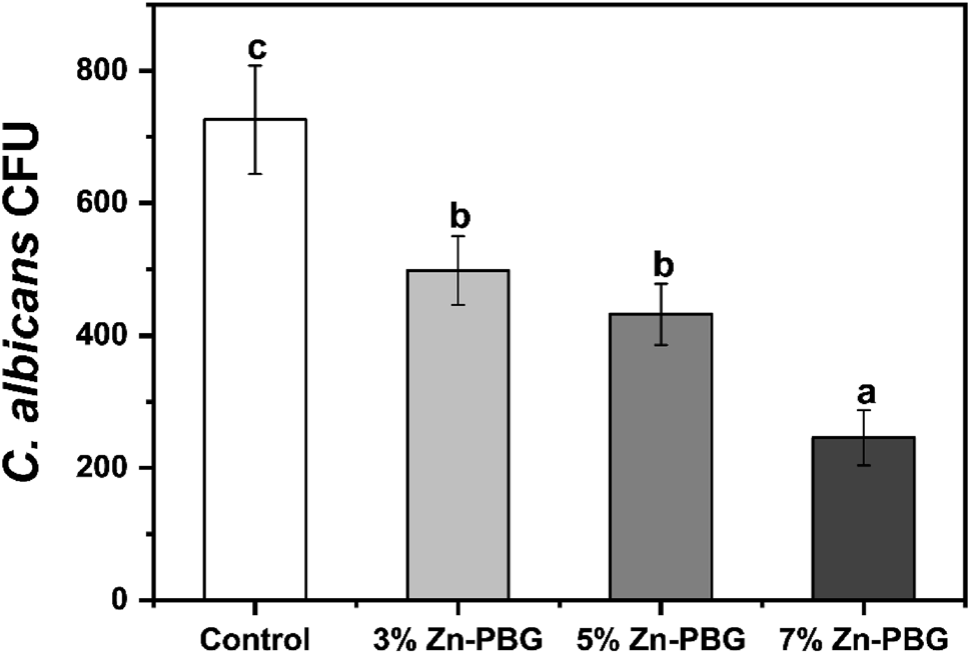
Colony-forming unit counts (CFU) of fungi on surfaces of various auto-polymerized acrylic resin samples: (**A**) control; **(B**) 3% Zn-PBG; (**C**) 5% Zn-PBG; (**D**) 7% Zn-PBG. Different lowercase letters above a bar indicate a significant difference at *p* < 0.05.

The SEM images demonstrated that the quantity of *C. Albicans* adhering to the surface was lower for the Zn-PBG groups than for the control groups (Fig. 4) and decreased with an increase in the content of Zn-PBG. Furthermore, in the quantitative analysis, log (CFUs/mL) for the control was significantly higher than that for the other Zn-PBG groups (*p* < 0.05) (Fig. 5).

### Ion release

**Figure 6:**
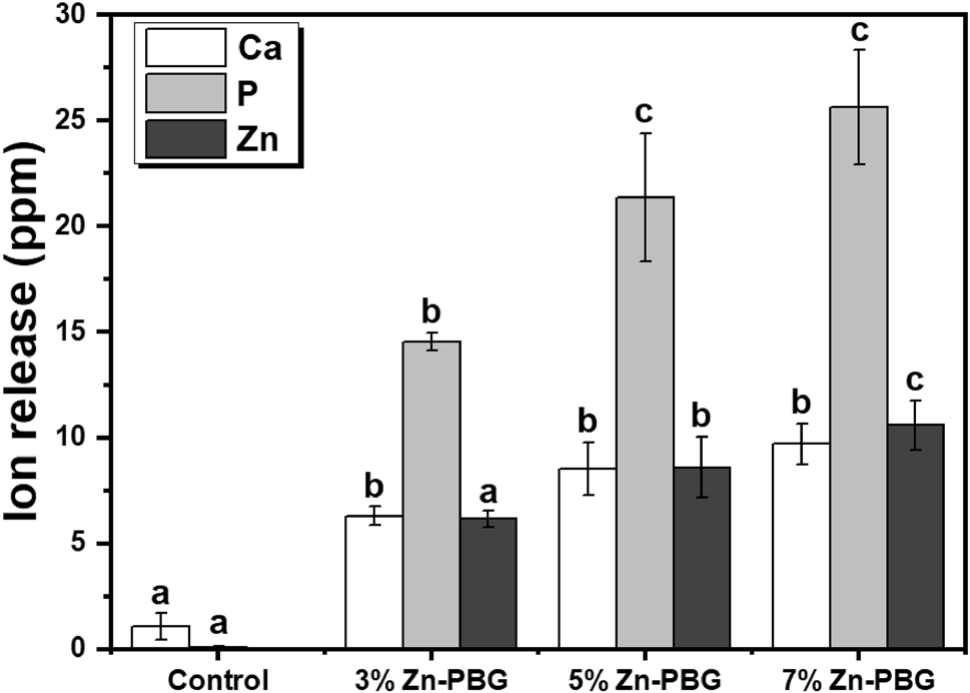
Concentration of ions (Ca, P, and Zn) released from each auto-polymerized acrylic resin sample. The same lowercase letter indicates that there is no statistical difference among the groups for the same ion.

The release of Ca, P, and Zn ions from each specimen is shown in Fig. 6. The results indicate an increase in the release of Ca and P ions with an increase in the amount of Zn-PBG. The release of Zn ions was not observed in the control group, in contrast to the experimental groups. There was a significant difference in the release of Zn ions from the 3% Zn-PBG group compared to the 5% Zn-PBG group (*p* < 0.05). Additionally, the maximum release of Zn ions was from the 7% Zn-PBG group, with a significant difference compared to the control group (*p* < 0.05). The concentrations of Ca and P ions for 3% Zn-PBG were significantly different from those for 5% and 7% Zn-PBG (*p* < 0.05).

## Discussion

Auto-polymerized acrylic resin has a long history of use and is one of the most developed and efficient dental materials. However, it has little or no antimicrobial activity. A recent systematic review detailed different antimicrobial additives for *C.*albicans^37^. The addition of agents such as silver zeolites, silver nanoparticles and silicate-based bioactive glass to denture base acrylic resin enhances their fungal resistance^37^. Therefore, the fabrication of an auto-polymerized acrylic resin with antimicrobial properties would be of great clinical benefit. The results of the present study agrees with that of the previous studies, which validate the use of Zn-PBG as a high-density filler for enhancing anti-microbial properties without a tangible loss of mechanical properties.

The specimens were prepared by the addition of Zn-PBG to auto-polymerized acrylic resin, and their physical and mechanical properties were evaluated. Flexural strength is an important property of dentures and there were no statistically significant differences in flexural strengths of 3% and 5% Zn-PBG compared to that of the control. The modulus of elasticity was also mostly unchanged. As the denture surface can be scratched and fractured under masticatory pressure, hardness is an important property^38^. The surface hardness of the auto-polymerized acrylic resin is also used to measure the resistance to the force applied during mastication; if the surface hardness is lowered, the stress distribution due to the masticatory force is not uniform^39^. There were no significant differences in the hardness of the Zn-PBG specimens compared to that of the control. The contact angle of the surface affects the adhesion of the bacteria and there were no significant differences in the contact angles of the experimental samples compared to that of the control. SEM observations also revealed no significant changes in morphology.

Physical strength, color tone, and surface shape of the denture base resin are affected by the use of denture cleaners or mechanical denture management. When the denture base is worn, the scratches induced by wear may roughen the surface and induce the accumulation of plaque and tartar^39^. Previous studies revealed that the presence of silver nanoparticles in resin resulted in high antifungal activity; however, deterioration, such as substantial changes in material properties or color, was not alleviated^30,40^. In contrast, the auto-polymerized acrylic resin containing Zn-PBG does not cause physical or mechanical differences from those of the control group. We argue that this was because Zn-PBG microparticles were synthesized by zinc modification of the phosphate-dominant bioglass^24^.

The number of *C. albicans* colonies attached to the surface of the control was very high, as confirmed by the SEM images; however, it gradually decreased as the content of Zn-PBG increased. The quantity of *C. albicans* attached to the surface was significantly lower in the 7% Zn-PBG group than in the control group. The results are similar to the findings of silver- and gallium-doped bioactive glass additives^37,41,42^. The effect against *C.albicans* observed in our study improved with Zn concentration, which is contrary to the observations when only nanoparticles were loaded. The authors reported a high variance and no surface attachment improvement with increasing concentration of silver nanoparticle^43^.

An analysis of the ion release results indicated that the contents of Ca, P, and Zn ions were significantly higher in the experimental groups than in the control group. Increasing the Zn-ion content was correlated with a decrease in the number of *C. albicans* isolates. Zn is an important mineral that plays a role in microbial inhibition^24^. Furthermore, the antibacterial activity of Zn ions and the binding action of Ca, P, Na, and Si ions stimulate the assimilation reaction^44^. These constructive effects depend on the concentration of Zn ions released; in previous studies, they were prominent at low concentrations and were exponentially suppressed as the zinc oxide content of the glass increased^29^. This is consistent with the results of this study, in which PMMA containing Zn-PBG exhibited an antimicrobial effect against *C. albicans*. However, the results of the present study reflect the effect of glass composition on ion release from a commercially designed PMMA system. Considering the above points, a modified resin matrix system could be developed that accounts for absolute ion release and related limiting dose while factoring possible adverse effects.

Our results can be utilized as basic data for the development of auto-polymerized acrylic resin with antimicrobial effects in the future. Furthermore, with an optimal concentration deduced from the results of the present study, future application with heat and microwave assisted denture base resins can be examined. However, long-term experimental studies are required to evaluate their clinical use. Cytotoxic tests and long-term studies of the durability of the antimicrobial effect must be performed, and the findings presented here should be confirmed by experiments conducted in vivo.

## Conclusion

In this study, an auto-polymerized acrylic resin containing Zn-PBG was fabricated, its physical and mechanical properties were measured, and its antimicrobial activity was confirmed. The main conclusions are as follows:

1. The addition of Zn-PBG did not result in loss of flexural strength, modulus of elasticity, hardness, or contact angle.
2. The *C. albicans* colony formation decreased as the Zn-PBG content increased.
3. The number of Ca, P, and Zn ions released increased as the Zn-PBG content increased.

Thus, incorporation of Zn-PBG into the auto-polymerized acrylic resin inhibited colonization by *C. albicans* without decreasing the overall physical and mechanical properties.

## Data Availability

The data will be made available by the corresponding author upon reasonable request.
